# Behavioral and neural measures of infant responsivity increase with maternal multisensory input in non‐irritable infants

**DOI:** 10.1002/brb3.3253

**Published:** 2023-10-02

**Authors:** Mary Lauren Neel, Arnaud Jeanvoine, Alexandra Key, Ann R. Stark, Elizabeth S. Norton, Lance M. Relland, Krystal Hay, Nathalie L. Maitre

**Affiliations:** ^1^ Department of Pediatrics & Neonatology Emory University School of Medicine & Children's Healthcare of Atlanta Atlanta, GA USA; ^2^ The Abigail Wexner Research Institute at Nationwide Children's Hospital Columbus, OH USA; ^3^ Emory University School of Medicine Atlanta, GA USA; ^4^ Department of Pediatrics & Neonatology Beth Israel Deaconess Medical Center & Harvard Medical School Boston, MA USA; ^5^ Northwestern University School of Communication Chicago, IL USA; ^6^ Department of Anesthesiology & Pain Medicine Nationwide Children's Hospital & The Ohio State University Columbus, OH USA

**Keywords:** electroencephalography, mother‐infant interaction

## Abstract

**Introduction:**

Parents often use sensory stimulation during early‐life interactions with infants. These interactions, including gazing, rocking, or singing, scaffold child development. Previous studies have examined infant neural processing during highly controlled sensory stimulus presentation paradigms.

**Objective:**

In this study, we investigated infant behavioral and neural responsiveness during a mother–child social interaction during which the mother provided infant stimulation with a progressive increase in the number of sensory modalities.

**Methods:**

We prospectively collected and analyzed video‐coded behavioral interactions and electroencephalogram (EEG) frontal asymmetry (FAS) from infants (*n* = 60) at 2–4 months born at ≥ 34 weeks gestation. As the number of sensory modalities progressively increased during the interaction, infant behaviors of emotional connection in facial expressiveness, sensitivity to mother, and vocal communication increased significantly. Conversely, infant FAS for the entire cohort did not change significantly. However, when we accounted for infant irritability, both video‐coded behaviors and EEG FAS markers of infant responsiveness increased across the interaction in the non‐irritable infants. The non‐irritable infants (49%) demonstrated positive FAS, indicating readiness to engage with, rather than to withdraw from, multisensory but not unisensory interactions with their mothers.

**Results:**

These results suggest that multisensory input from mothers is associated with greater infant neural approach state and highlight the importance of infant behavioral state during neural measures of infant responsiveness.

## Graphical abstract

In this study, we investigated infant behavioral and neural responsiveness during a mother–child social interaction during which the mother provided infant stimulation with a progressive increase in the number of sensory modalities. As the number of sensory modalities progressively increased during the interaction, infant behaviors of emotional connection in facial expressiveness, sensitivity to mother, and vocal communication increased significantly. Non‐irritable infants (49%) demonstrated positive FAS, indicating readiness to engage with, rather than to withdraw from, multisensory but not unisensory interactions with their mothers.

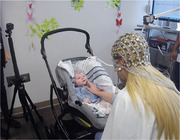

## 1 BACKGROUND

Parent–infant interactions are foundational to child development (Hall et al., 2015; Maupin & Fine, 2014; Neel et al., 2018; Treyvaud et al., 2016). Measurement of infant responsivity during complex parent–infant interactions is critical to understanding and supporting optimal child development. To assess infant responses during mother–infant interactions, previous studies have used behavioral coding of infant facial expressions, vocalizations, and/or overall clarity of visible cues (Hane et al., 2018; Kang et al., 1995; Reynolds et al., 2014; White‐Traut et al., 2013). That work showed that infants look to their mother more often when she displays both auditory and visual expressions than when she displays auditory or visual expressions alone, indicating that infants are more responsive to maternal multisensory, than unisensory, input (Vaish & Striano, 2004). For mother–preterm infant dyads in the intensive care unit, interventions that include multisensory components, such as auditory, tactile, visual, and vestibular (ATVV) stimulation, are associated with improved infant clarity of cues on behavioral coding (White‐Traut et al., 2013). These studies support the possibility that infant responsivity increases with maternal multisensory input and can be measured using behavioral coding (behavioral responsiveness). However, the sensitivity of infant facial behavioral coding to changes in response following an increasing number of modalities added by the mother in a structured, layered multisensory social interaction is unknown. We predict that facial coding measures would reflect increased infant positive emotion to the mother's engagement with a progressive addition of visual, tactile, and auditory inputs.

Because behavioral facial expressions are still developing in the first 4 months, behavioral coding in early life may not reflect the full extent of infant responses (Lewis, 2011a, 2011b; Lilley et al., 1997; Relland et al., 2019). To address this challenge and to add to the literature on electroencephalogram (EEG) in infants (Anaya et al., 2021), we used EEG measures as an objective marker of infant responsivity to increasing multisensory stimulation (neural responsiveness). EEG has been used to examine brain responses to multisensory stimulation. Previous reports noted audio‐visual stimulation increased infant neural responses over the frontotemporal scalp regions compared to auditory stimuli alone (Hyde et al., 2010). Similarly, synchronous audio‐visual stimulation was associated with greater amplitudes in the parietal locations and a reduction in amplitude of the temporal electrodes compared to asynchronous stimulation or visual stimuli alone, suggesting greater attention and stimulus‐processing efficiency for multisensory stimulation (Reynolds et al., 2014).

Previous work has examined specific infant EEG event‐related potential (stimulus‐linked) components that are associated with attention (P2 component), response to face (N290 component), or stimulus familiarity (Nc component) ( Amodio, 2010; Carver et al., 2003; Conte et al., 2020). However, component analysis is designed for event‐related potential paradigms rather than spectral analysis of longer events. In the context of a mother–child interaction, maternal sensory stimulation likely elicits infant emotional responses in addition to the processing of sensory stimuli. Frontal asymmetry of alpha‐band EEG signals has been extensively studied and established as an informative measure of emotional reactivity in infants and adults (Anaya et al., 2021; Coan & Allen, 2004; Coan et al., 2006; Davidson, 1993; Gartstein, 2019; Howarth et al., 2016; Licata et al., 2015). Frontal asymmetry is more pronounced during procedures designed to elicit emotional responses compared to resting protocols (Coan et al., 2006). Thus, frontal asymmetry is a well‐suited and convenient marker for paradigms evaluating infants during interactions containing a combination of emotion, sensory stimulation, and attention.

Even in infants, left‐shifted frontal asymmetry seems to indicate approach and right‐shifted frontal asymmetry seems to indicate withdrawal (Anaya et al., 2021; Davidson, 1993). In infants, greater left than right frontal neural activity (lower alpha power over the left hemisphere/left‐shift) has been associated with strangers making happy (vs. sad) faces (Davidson, 1992; Davidson & Fox, 1982) and with infants who reach for their mothers during an approach interaction (Fox & Davidson, 1987). In addition, higher infant‐positive affect was associated with greater left frontal asymmetry in the context of a mildly stressful interaction (Gartstein, 2019). Conversely, greater right frontal activity (right‐shift) has been associated with infant‐negative emotional reactions, such as crying in response to maternal separation (Davidson & Fox, 1989). These results support the relationship between frontal asymmetry and emotional reaction, even in infants, (Coan & Allen, 2004).

We hypothesized that infants’ more positive engagement in response to an increasing number of infant‐directed maternal sensory stimuli would be reflected by greater left than right frontal activity during this combined sensory and emotion‐eliciting interaction paradigm (Chen et al., 2016; Coan et al., 2006; D. C. Hyde et al., 2010; Maitre et al., 2017, 2020; Therien et al., 2004). We predicted that frontal asymmetry in infants would show changes in activation patterns as the mother added a greater number of concordant sensory stimuli to the interaction.

### 1.1 Present study

Our study is the first to evaluate infant neural responsiveness within the context of a mother–child interaction during which the mother provides stimulation with a progressively increasing number of sensory modalities. Previous studies have focused on neural sensory stimulus processing provided by carefully standardized stimulus presentation, for example, visual stimulation with a 10.5‐cm circle, auditory stimulation with a 150 Hz tone, or highly standardized 1700‐ms videos with visual and/or auditory stimuli (Hyde et al., 2010; Reynolds et al., 2014). To build on this previous work and test the validity of our experimental paradigm and EEG variables of infant neural responsiveness, we first performed behavioral coding of the mother and infant during the interaction. We aimed to test the association of infants’ frontal asymmetry with accepted behavioral facial coding while accounting for mother and infant characteristics that could be associated with tendencies for mothers (baseline bondedness) and infants (irritability) to respond to each other (Feldman et al., 2007; Nolvi et al., 2016). By accounting for these tendencies, we tested the possibility that the brain‐behavior associations are due to the immediate effects of the mother and child on each other. We hypothesized that as the mother added more sensory cues to the interaction: (1) infant behavioral responsiveness, as measured by facial coding scores, would reflect greater connection and engagement and (2) neural responsiveness, measured by EEG, would reflect increasingly greater left than right frontal activity.

## 2 METHODS

### 2.1 Participants

We performed a prospective study of infant response to multisensory input from their mother. Eligible infants were born at ≥34 weeks gestation, corrected age (CA) 2 to 4 months postterm at the time of the study, and were healthy, with no history of birth complications (see demographics in Table 3). We selected this age range because developmental milestones in socio‐emotional engagement, including visual attention, social smile, and neck control to orient to external stimuli, are visible on video coding. We excluded infants who had vision or hearing abnormalities, were unable to track visually 10 degrees from midline on either side, or had direct and/or maternal exposure to analgesics or sedatives within 72 h of the study. The study was approved by the Nationwide Children's Hospital Institutional Review Board, and written informed consent was obtained from the parent(s) of each infant.

### 2.2 Procedures

We collected dual EEG recordings of mother and infant throughout the interaction; here, we report infant EEG. We video‐recorded this interaction and subsequently coded infant behaviors. Finally, we assessed mother–infant bondedness via the Mother‐to‐Infant Bonding Scale (MIBS) questionnaire (Taylor et al., 2005) and maternal depression using the Edinburgh Postnatal Depression Scale (EPDS) (Cox et al., 1987).

**FIGURE 1:**
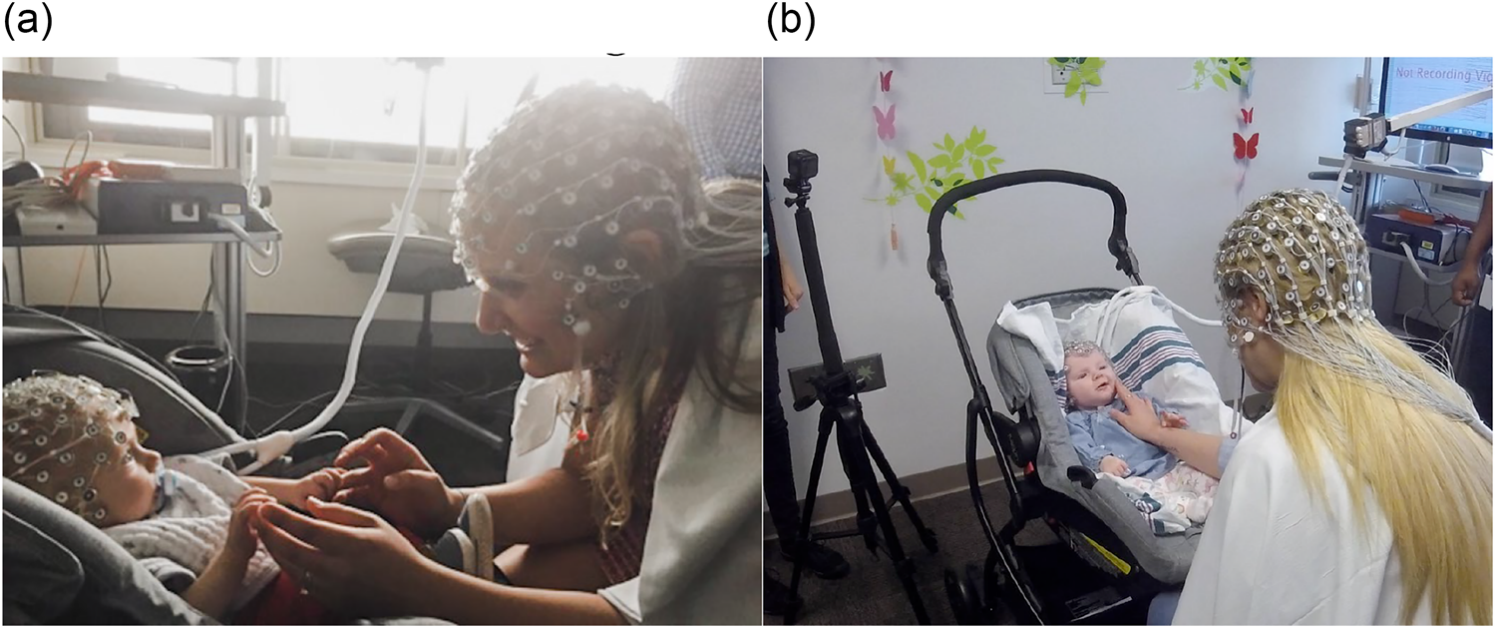
(a) Mother and infant study set up. (b) Mother and infant output, generated by two cameras and two electroencephalogram (EEG) systems for behavioral and neural coding.

Mother–infant dyads completed a structured naturalistic interaction during which the mother provided infant‐directed sensory inputs. This consisted of a series of four distinct actions, each performed in duplicate by the mother in her own manner, and sequenced to add progressive layers of sensory scaffolding from mother to infant (Table 1, Table 2). Action 1 served as mother–infant baseline and actions 2–4 were intended to promote mother–infant engagement. Mothers received scripted instructions on how to perform each action and were monitored and guided by the examiner for fidelity to the script. Each action lasted 10 s and was separated by a 15‐s pause to prevent anticipation or habituation. One run comprised eight actions and was cycled twice with a 2‐min break between runs. The entire two‐run procedure took approximately 10 min, while the mother and infant sat across from each other with their faces at optimal focusing distance for the infant (24–30 in.) (Figure 1). An examiner behind the infant cued the mother when to start and stop each action (Table S1).

**TABLE 1:** Sequence of actions comprising multisensory interaction procedure. Each action in the interaction procedure added a layer of sensory scaffolding from mother to infant.

Action (each 10 s)	Description
1	Mother looks above baby
2	Mother smiles warmly at baby
3	Mother smiles and affectionately touches baby's cheek
4	Mother smiles and affectionately touches baby's cheek and says with emotion “I love you *baby's name*”

**TABLE 2:** Definition of terms.

Term	Definition
Action	Each 10‐s scripted and prompted action performed by the mother; see Table 1
Trial	Includes actions 1–4; four trials total in paradigm; the signals of the four trials are averaged using analytic tools (cross‐correlation) to align the start of each action across trials
Run	Eight actions; two trials; two runs total in the paradigm
Segment	EEG product of action

### 2.3 EEG recording, processing, and analysis of frontal asymmetry score

EEG was recorded during the entire multisensory interaction using a high‐density array of 128 electrodes embedded in soft sponges on an appropriately sized cap (Hydrocel Sensor Net, EGI, Inc., Eugene, OR), with the reference electrode at the vertex and a sampling rate of 1000 Hz (Net Station v. 5.2.0.2; EGI, Inc., Eugene, OR). We used an auditory cue (countdown) to signal the change in activity and mark the corresponding EEG periods. The mark‐up was further verified based on a concurrent video recording.

We ensured that impedance was <50 kΩ at all electrodes before the start of the paradigm to minimize the change of unusable electrode data. EEGs were processed using Net Station tools (Figure 3). The recorded signal was preprocessed using a notch filter (60 Hz), a bandpass filter (Butterworth, order = 1) 0.3–40 Hz (Debnath et al., 2020), and re‐referenced to the average reference. An action segment was excluded if during the recording >35 electrodes had unusable data. For subjects with ≤ 35 unusable electrodes, any noisy data were corrected using interpolation from surrounding electrodes and standard algorithms in Net Station.

**FIGURE 2:**
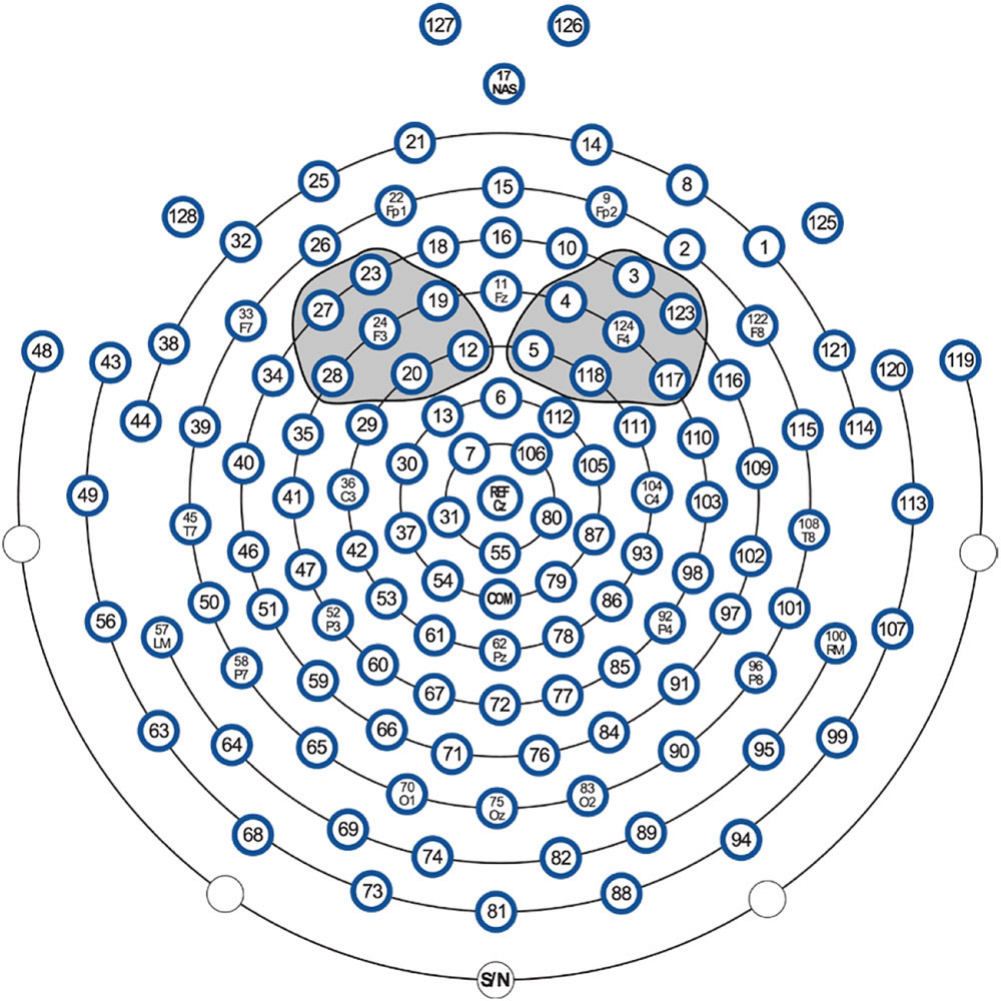
Electrodes on electroencephalogram (EEG) net with those surrounding F3 and F4 locations circled in gray.

**FIGURE 3:**
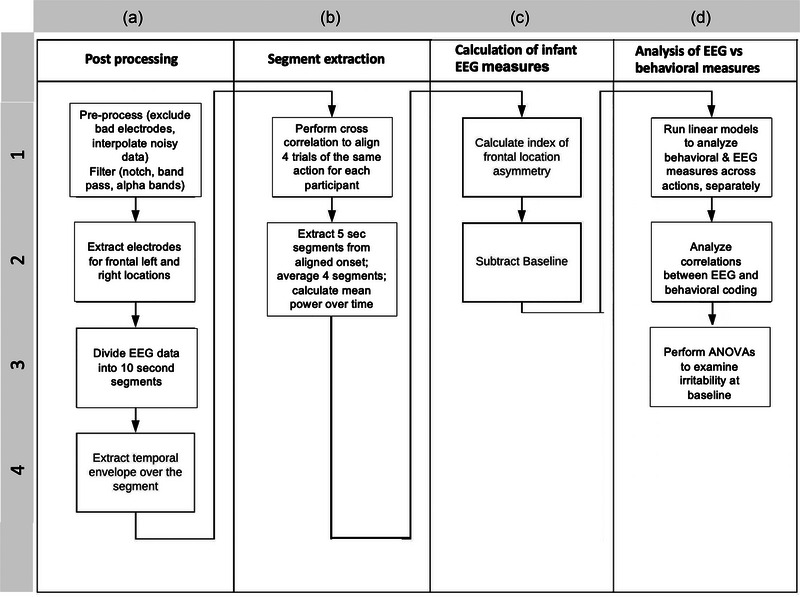
Flow diagram of methodology. EEG, electroencephalogram.

Infant alpha band (6–9 Hz) data were extracted based upon previous reports (Bell, 2002; Marshall et al., 2002) (Figure 3, A1 and A2) and averaged across the seven electrodes surrounding F3 and F4 locations (Figure 2). EEG data were analyzed after the elimination of amplitudes > 120 μV to account for artifacts, including eye blinks and movement. Of note, the quality of the EEG data on the continuous recording was similar between the irritable and non‐irritable infants.

The analysis focused on the alpha signal for each action, quantified within the individual epochs from 0 to 10,000 ms after the action onset trigger (Figure 3, A3). Thus, four epochs were analyzed for each action: two trials for each of the two runs. We calculated the envelope of the signal to measure the positive value of the amplitude of the oscillation during each trial (Figure 3, A4). Envelopes of the signal in the left and right frontal electrodes (Figure 2) were calculated using a Hilbert transform (Feldman et al., 2009) from the *scipy* module (Virtanen et al., 2020) in python 3.7 (Van Rossum & Drake, 2009).

In different iterations of the same action, mothers demonstrated natural variations in timing between hearing the command to perform and executing the action, thus impacting infant neural response timing. To compensate for these timing variations and to allow for averaging of different iterations of the same action, we used a cross‐correlational analysis of the global field power (filtered on alpha‐band) across the 10,000 ms of the response to each action within an individual infant's trials. While this type of analysis is sometimes used between individuals to compare their responses, in this study, we do not use it for such a purpose. Instead, we use this analysis within a single individual. Here, cross‐correlational analysis is the only data‐driven technique to average the phase of signal alignment for multiple trials of the same action in the same participant. This analysis aligned all responses to the same action, for the same participant, to their peak maxima, compensating for timing delays and allowing for cross‐correlation of the envelope of the signal (Semmlow, 2012). By running EEG data for each individual's action segments through correlation in the *numpy* module (Harris et al., 2020), we obtained the maximum likelihood of alignment between all four measurements of the same action (Figure 3, B1). For each aligned action, we calculated the mean alpha power of the signal envelope in F3 and F4 clusters, across the 5000‐ms action segment centered on the aligned peak (Figure 3, B2). Given that each action was 10,000 ms and based upon video recordings of infant responses, we accounted for a 2000‐ms delay in infant response after action onset due to the time it took the mother to perform the action. This delay allowed us to capture the true infant electrophysiological reaction to the maternal stimulus. To ensure no overlap between one action and the following action, we used the 5000 ms after the first 2000 ms.

We calculated the frontal asymmetry score (FAS) for each action for each infant as the difference between left and right frontal alpha power of the signal envelope as a measure of neural activity (Davidson et al., 1990) using the equation: FAS = (F3−F4)/(F3+F4) (Allen et al., 2004; Coan & Allen, 2004). This formula yields an index value between −1 and 1. A value near 0 indicates similar alpha power amplitudes over the left and right hemispheres. A positive or negative value indicates lateralization to the left or right side, respectively (Figure 3, C1). We subtracted the infant's FAS amplitude of action 1 (which corresponds to the baseline) from each of the following actions (Figure 3, C2), resulting in three FAS values for each infant.

### 2.4 Behavioral coding

We video‐recorded the mother–child session, with the frame focused on the infant's face. Infant behavioral responses to each action were coded in the domains of facial expressiveness, sensitivity to mother (responsiveness to mother's expressed emotions), and vocal communication using the standard three‐point scale from the Welch Emotional Connection Scale (WECS) (Hane et al., 2019, 2019). Higher scores on the WECS indicate a greater emotional connection to the mother. The WECS has been well‐validated as a measure of mother–infant behavioral responsiveness and correlates with infant biobehavioral measures and 3‐year behavioral outcomes (Frosch et al., 2019; Hane et al., 2019). Per standardized coding methods described in the literature, we coded infant behavior as a 1 when the behavior in the domain was absent, a 2 for mixed behaviors, and a 3 for clearly present behaviors (Hane et al., 2018, 2019). A 0 was coded for sensitivity to the mother if the child was markedly drowsy. Raw coding scores were averaged across the four iterations of each action to generate final scores for each domain and action (Table S2). After establishing inter‐rater reliability of >85% among three coders for all infant domains on a subset of 10 videos, we randomly divided video‐recordings among the three coders. The primary coder (KH) reviewed additional videos (>50%) as a second coder to ensure inter‐rater reliability.

Coders also classified the infant's emotional state for each action using the emotional state scale of the Hammersmith Infant Neurological Exam (Dubowitz et al., 1999). This classification considered an infant as irritable if he/she was not consolable at any point during the interaction or if he/she needed consoling more than once during the interaction. Infants who were only irritable when initially approached, neither happy nor unhappy, or happy and smiling were classified as not irritable (Wakschlag et al., 2015). WECS data for any action during which the infant's behavioral state was “inconsolable” were eliminated, as inconsolability confounded the ability to code facial expressions.

### 2.5 Maternal factors

Because maternal factors may influence interactions with the infant, we administered the MIBS to assess maternal perception of bondedness with her infant (Taylor et al., 2005) and the EPDS (Cox et al., 1987) to screen for maternal depression given the postpartum interval during which we conducted our study. The MIBS test range is 0–24. Lower scores indicate greater maternal perception of bondedness with an “at risk” threshold ≥2 (Bienfait et al., 2011; Taylor et al., 2005; Wittkowski et al., 2007). The EPDS test range is 0–30 (Cox et al., 1987). Higher scores indicate greater concern for depression with a conservative “at risk” threshold of ≥10 (Bienfait et al., 2011; Taylor et al., 2005).

### 2.6 Analysis plan

We used a linear model to test the hypothesis that facial behavioral coded WECS scores (continuous variable) would increase across actions, as more sensory modalities were added to the interaction. Similarly, we used a linear model to compare infant FAS (continuous variable) across actions to test our hypothesis that infants would demonstrate increasingly more left frontal activity as actions (Figure 3, D1). We then used Pearson correlations to examine associations between infant behavioral responsiveness (video‐coded infant WECS scores) and neural responsiveness (infant FAS) across the mother–infant interaction procedure (Figure 3, D2). To account for the possibility that infant irritability and maternal bondedness might impact results, we computed interaction terms of the interaction accounting for irritability to determine if subgroup analysis between irritable and non‐irritable infants was justified (Figure 3, D3). The same analytic plan was conducted using interaction terms of the interaction accounting for maternal bondedness to determine if subgroup analysis was justified.

## 3 RESULTS

### 3.1 Demographics and summary statistics

We prospectively enrolled 60 mother–infant dyads between July and December 2017. Although all completed the protocol, data from nine subjects were excluded from analysis due to poor quality or missing EEG data. Of the 51 infants included in the analysis, the median gestational age at birth was 39 weeks and the postnatal age was 3 months at the time of the study. Only 3% of emotional states were coded as inconsolable. Irritable/non‐irritable scores and WECS data were missing in two infants due to malposition and were excluded from analyses contingent upon these data. The median maternal MIBS score was 1 and the median maternal EPDS score was 3. Since no mothers met the EPDS threshold for depression (Table 3), no further analyses included maternal depression.

**TABLE 3:** Population characteristics.

(A) Population demographics		
Infant characteristic (*N* = 51)	*N*	%, unless noted*
Female	25	49
GA weeks (median, IQR)*	39	(39, 40)
Corrected age days (median, IQR)*	92	(75, 113)
Irritable (*N* = 49 for video‐coding)	25	51
Race		
White	39	76
Black or African‐American	3	6
Asian	2	4
More than one race	7	14
Ethnicity		
Hispanic	3	6
Not Hispanic	44	86
No answer	4	8
Maternal education		
Partial college or trade school	6	12
College graduation	19	37
Graduate education	26	51

(B) Maternal scores		
Maternal score (*N* = 51)	*N*, unless noted*	%, unless noted*
MIBS score (median, IQR)*	1	(0, 2)
0	18	35
1	14	27
2	12	24
3	3	6
4	1	2
5	3	6
EPDS score (median, IQR)*	3	(1,6)

(C) Infant WECS scores across actions, *N* = 49				
Action	1	2	3	4
Welch facial expressiveness (median, IQR)	1.3 (1, 2)	1.5 (1, 2.3)	2 (1.5, 2.3)	2 (1.5, 2.5)
Welch sensitivity (median, IQR)	2 (1.5, 2.8)	2.6 (2, 3)	2.5 (2.3, 3)	3 (2, 3)
Welch vocalizations (median, IQR)	2 (2,2)	2 (2, 2)	2 (2, 2.3)	2 (2, 2.5)

Abbreviations: EPDS, Edinburgh Postnatal Depression Scale; MIBS, Mother‐to‐Infant Bonding Scale; WECS, Welch Emotional Connection Scale.

### 3.2 Infant behavioral responsiveness to multisensory interaction

**FIGURE 4:**
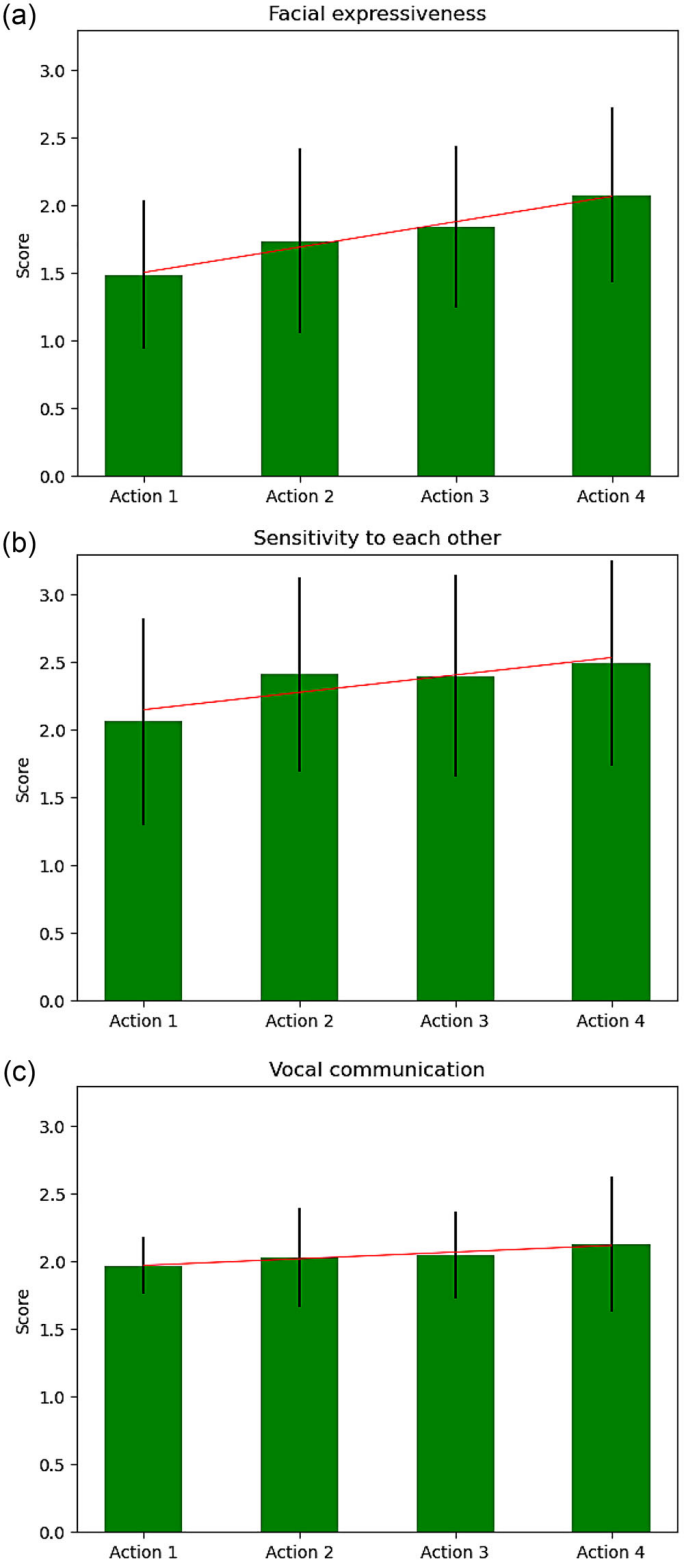
Infant Welch Emotional Connection Scale (WECS) scores' mean (bar graph) and standard error (black vertical lines) for facial expressiveness (a), sensitivity to mother (b), and vocal communication (c) increased across actions as mother added more layered sensory input throughout interaction.

As sensory modalities were added across the multisensory interaction, video‐coded infant WECS (0 to 3) increased in facial expressiveness (*r* = .32, *p* < .001), sensitivity to mother (*r* = .19, *p* = .009), and vocal communication (*r* = .15, *p* = .038) (Table 3, C and Figure 4).

### 3.3 Infant neural responsiveness to multisensory interaction

**FIGURE 5:**
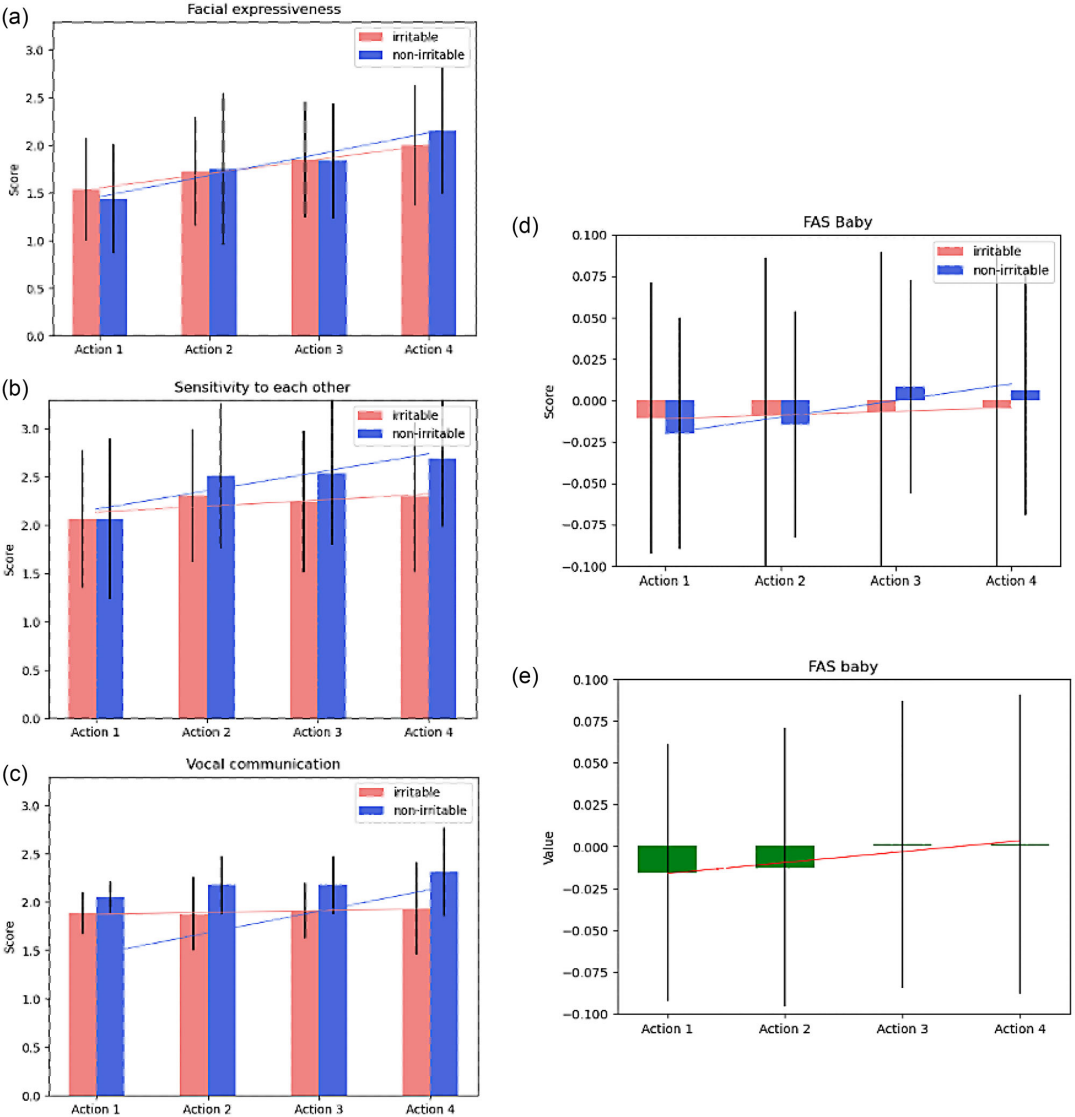
All infant Welch Emotional Connection Scale (WECS) scores for facial expressiveness (a), sensitivity to mother (b), vocal communication (c), and infant frontal asymmetry score (FAS) values (d) increased across actions as the mother added more layered sensory input in non‐irritable (blue) but not irritable (red) infant groups. While infant FAS visually trended up across actions as the mother added more layered sensory input, the difference was not statistically significant (e).

In contrast to behavioral data, infant FAS across actions for the entire cohort did not have a statistically significant association with the increasing number of sensory modalities introduced by the mother (*r* = .11, *p* = .123) (Figure 5e).

### 3.4 Infant irritability is associated with group differences in response to mother's actions

To examine the effect of infant irritability on mother–infant interactions, we compared infant WECS scores (Figure 5a–c) and FAS values (Figure 5d) across actions between irritable and non‐irritable infant groups. We found that all WECS scores significantly increased across actions for non‐irritable infants (WFAC *r* = .36, *p* < .001; WSENS *r* = .28, *p* = .007; WVOC *r* = .27, *p* = .008), but not for irritable infants (WFAC *r* = .28, *p* = .007; WSENS *r* = .10, *p* = .356; WVOC *r* = .06, *p* = .574) (Figure 5a–c). Similarly, infant FAS values were significantly increased across actions in non‐irritable infants (*r* = .21, *p* = .043), but not in irritable infants (*r* = .04, *p* = .713) (Figure 5d).

### 3.5 Infant group differences in response to mother's actions are associated with mother bondedness scores

**FIGURE 6:**
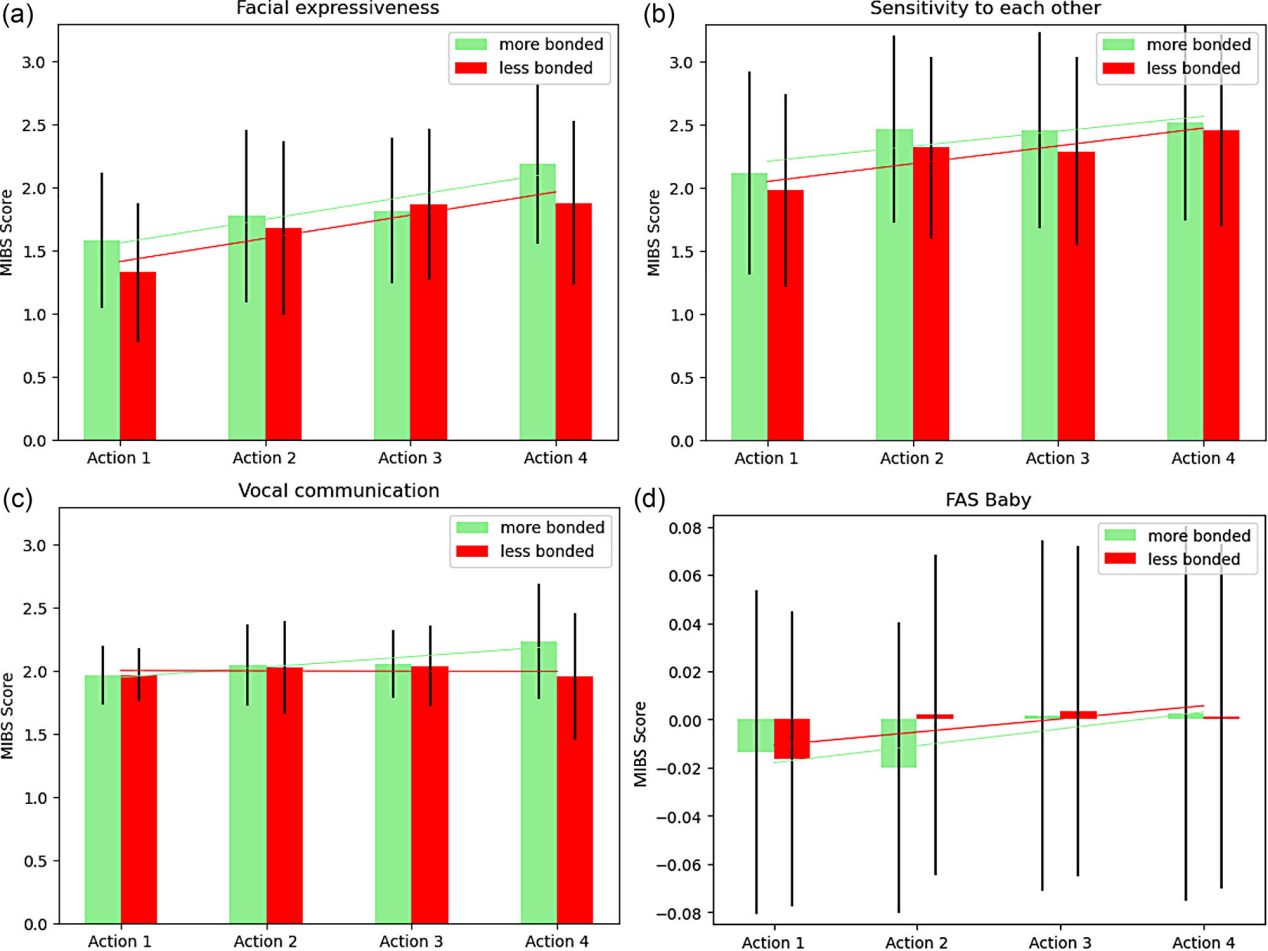
Infant Welch Emotional Connection Scale (WECS) scores for facial expressiveness (a), sensitivity to mother (b), vocal communication (c), and infant FAS values (d) across actions by more (green) and less (red) bonded mother groups. The more bonded group showed a significant increase across actions for facial expressiveness (a) and vocal communication (c). The less bonded group showed a significant increase across actions only for facial expressiveness (a).

Because maternal factors influence mother–infant interactions, we also investigated group differences in infant responses based on maternal factors such as bondedness. To examine the impact of maternal bondedness on infant responses across this interaction, we compared infant WECS scores (Figure 6a–c) and FAS values (Figure 6d) across actions between infant groups with mothers who were more bonded or less bonded. In keeping with previous work, mothers who scored 0 or 1 on the MIBS comprised the more bonded group, while infants whose mothers scored ≥2 on the MIBS comprised the less bonded group (Bienfait et al., 2011; Taylor et al., 2005; Wittkowski et al., 2007). Both groups showed WECS scores increasing significantly across actions for facial expressiveness (more bonded *r* = .33, *p* < .001; less bonded *r* = .32, *p* = .006), while only the more bonded group showed a significant increase across actions for vocal communication (more bonded *r* = .27, *p* = .003; less bonded *r* = −.01, *p* = .946). No significant differences were found for sensitivity to mother (more bonded *r* = .17, *p* = .067; less bonded *r* = .22, *p* = .058) or infant FAS (more bonded *r* = .012, *p* = .198; less bonded *r* = .10, *p* = .405) across actions for either group (see Figure 6a–d).

### 3.6 Comparing infant behavioral and neural responsiveness across multisensory interaction

**FIGURE 7:**
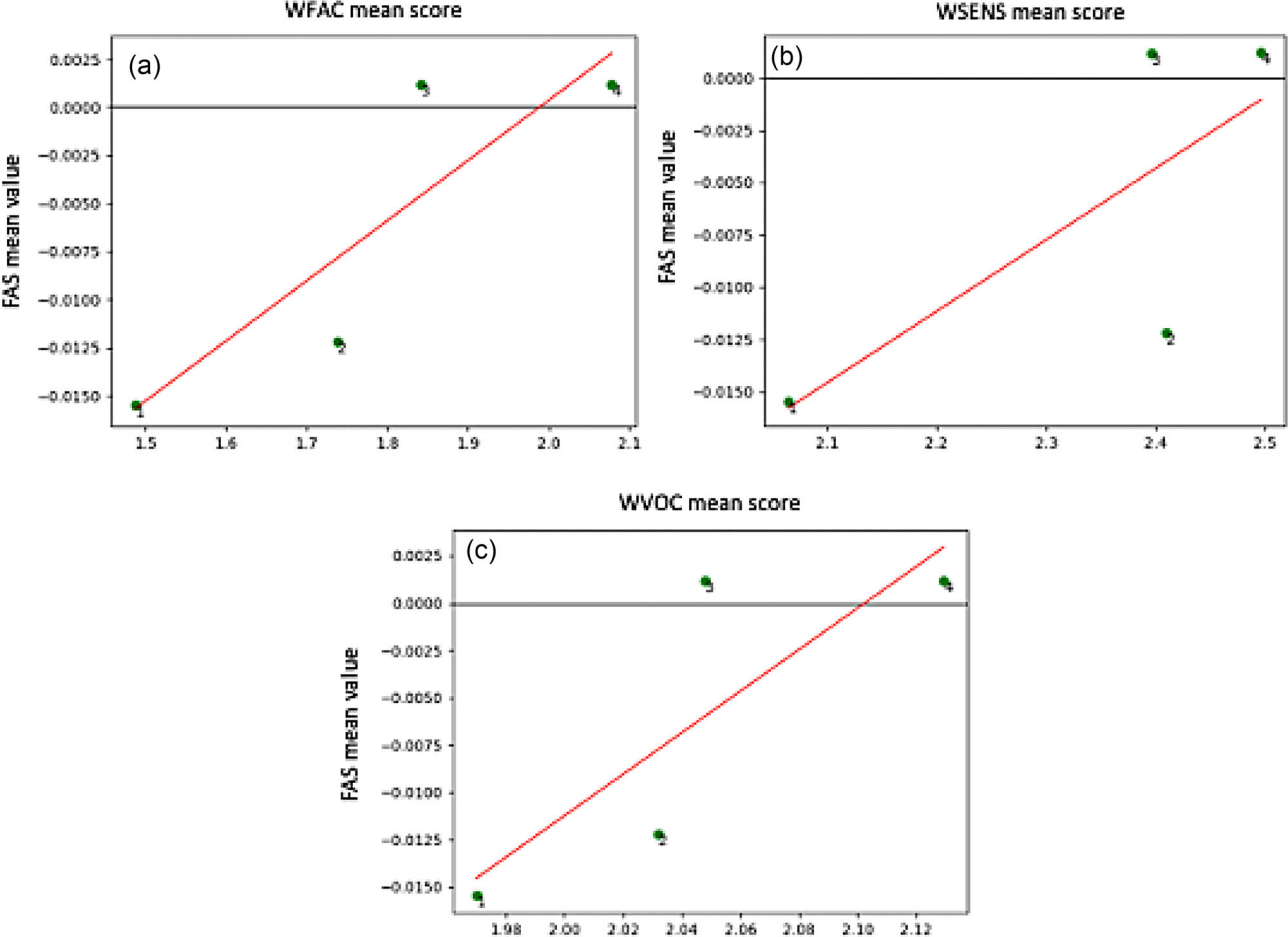
Correlations between grand average infant frontal asymmetry score (FAS) values and infant Welch Emotional Connection Scale (WECS) scores for facial expressiveness (a), sensitivity to mother (b), and vocal communication (c) for these *n* = 51 infants. Actions are noted as 1, 2, 3, and 4. For actions 1 and 2, infant FAS/WECS correlations were negative, while for actions 3 and 4, correlations were positive.

When we compared grand average infant WECS scores and FAS values by actions, for all correlations the WECS and FAS for actions 3 and 4 were more positively correlated than for actions 1 and 2 (WFAC/FAS *r* = .87, *p* = .123; WSENS/FAS *r* = .74, *p* = .259; WVOC/FAS *r* = .82, *p* = .179) (see Figure 7). When the baseline (Action 1) was subtracted from subsequent actions, correlations remained the same.

### 3.7 Comparing infant behavioral and neural responsiveness across interaction accounting for infant irritability and maternal bondedness

To evaluate the effect of infant irritability on the WECS and FAS across actions, we tested the interaction terms of WECS facial, sensitivity, or vocal communication and irritable status, which showed significant differences for interaction across actions (WFAC: *r* = .17, *p* = .020; WSENS *r* = .15, *p* = .045; WVOC *r* = .13, *p* = .066). These significant or nearly significant results allowed us to examine correlations between WECS/FAS by irritable and non‐irritable groups.

**FIGURE 8:**
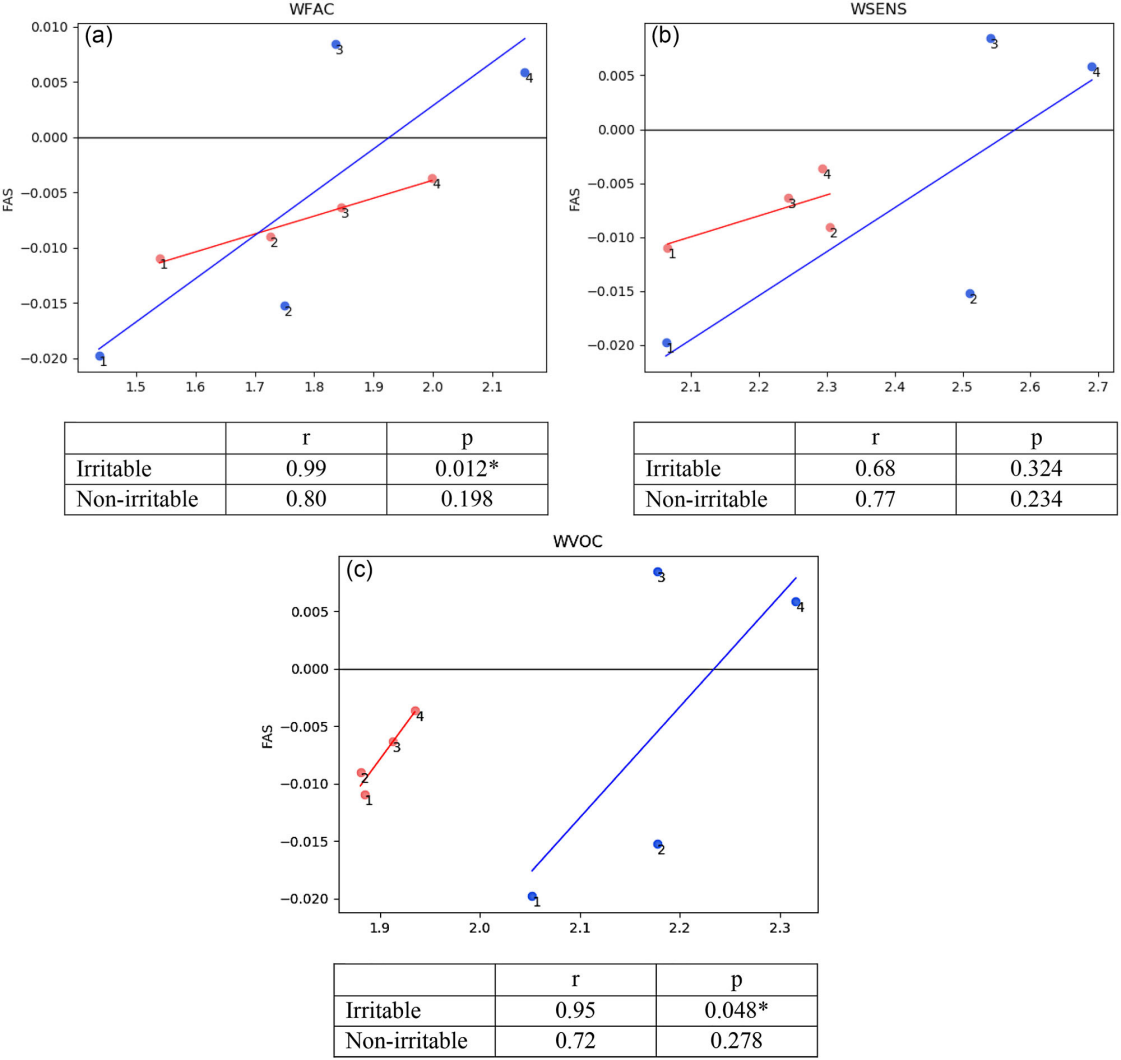
Correlations between grand average infant frontal asymmetry score (FAS) values and infant Welch Emotional Connection Scale (WECS) scores for facial expressiveness (a), sensitivity to mother (b), and vocal communication (c) divided by irritable (red) and non‐irritable (blue) groups. Actions are noted as 1, 2, 3, and 4. For the non‐irritable group, infant FAS/WECS correlations were negative for actions 1 and 2 and positive for actions 3 and 4. For both irritable and non‐irritable groups, the slopes of the correlations trended toward more positive across actions. * Significant *p* value of ≤ .05.

For both irritable and non‐irritable infant subgroups, the slopes of the FAS/WECS correlations trended toward more positive across actions. One pattern of note is that for non‐irritable infants, correlations for actions 1 and 2 were negative while for actions 3 and 4, the correlations were positive. For irritable infants, all correlations were negative (see Figure 8).

To further analyze correlations between WECS scores and FAS by more or less bonded groups, we calculated an interaction term of WECS facial, sensitivity, or vocal communication and bondedness status across actions. No significant differences were found for interaction terms by bondedness status (WFAC *r* = −.08, *p* = .285; WSENS *r* = −.09, *p* = .221; WVOC *r* = −.11, *p* = .147). Thus, we do not report further analysis of correlations between grand average WECS and FAS by more or less bonded groups as this group analysis was not justified.

## 4 DISCUSSION

Our study is the first to test a measure of infant neural responsiveness (FAS) with accepted behavioral facial coding throughout a scripted, yet naturalistic, mother–infant multisensory interaction. Previous studies of infant neural responsiveness use highly controlled sensory stimulus presentation paradigms (Hyde et al., 2010; Reynolds et al., 2014). In this study, we also considered and accounted for infant irritability and maternal bondedness throughout our experimental procedure as these factors could impact dyadic interaction and subsequent infant responsiveness.

Layering sensory input during maternal–infant interaction appears to increase infant engagement in the interaction. For the entire cohort, behavioral measures of infant responsiveness (WECS) increased across the interaction as the mother progressively increased the number of modalities used. Neural responsiveness followed the same trend but did not reach significance, likely due to the large variation in infant response in the limited number of study participants.

However, when we examined irritable and non‐irritable subgroups, we observed key differences. All behavioral and neural measures of infant responsiveness increased across actions in non‐irritable infants, but not in irritable infants. A possible explanation is that non‐irritable infants can be successfully engaged by layering sensory input during interactions with their mothers, while irritable ones cannot. The slopes of the correlations between infant WECS and infant FAS across actions became more positive across the interaction sequence, indicating that infant behavioral and neural responsiveness were increasing with additional maternally provided sensory modalities. Furthermore, in non‐irritable infants, this correlation became positive in Actions 3 and 4 (multisensory actions) as infant FAS transitioned from negative to positive (right to left frontal asymmetry).

### 4.1 Infant measures of responsiveness are highly associated with infant behavioral state during the mother–infant interaction

Early visual, tactile, and auditory cues are central to mother–infant communication and responsiveness during the first months of life. Early interventions that include supportive sensory components have been associated with improved infant‐responsive behaviors toward mothers (Beebe et al., 2018; White‐Traut & Nelson, 1988; White‐Traut et al., 2013). Infants in the first months of life respond with more eye contact, vocalizations, and smiling to face‐to‐face encounters that include a tactile component, along with visual and vocal cues (Peláez‐Nogueras et al., 1996). In many cases, parents intuit the importance of layered sensory input. For example, parents of deaf infants play “vocal games” with their infants that incorporate visual and tactile cues, and these deaf infants vocalize as much as their hearing counterparts (Koester et al., 1998).

Consistent with our hypotheses, all infant behavioral and neural measures of responsiveness increased across actions. However, this hypothesis proved true only in the non‐irritable infants, a finding we did not specifically predict. Irritability in infants is a complex conglomeration of nature and nurture(Crockenberg & Smith, 1982), and young children demonstrate various trajectories in their degree of irritability over time (Wiggins et al., 2014). State is a temporary characteristic, whereas trait indicates a persistent tendency toward a characteristic. For example, an infant might exhibit irritability in the moment (state), but not have a general tendency toward irritability (trait). However, this issue is complicated because an infant with a trait‐like tendency toward irritability (irritable temperament) might experience temporary irritable states more often.

The importance of the infant behavioral state during the mother–infant interaction highlights interesting distinctions between irritable and non‐irritable infant responses and evaluation. As a result, infant behavioral state, especially in artificial environments such as laboratory settings, should be accounted for when studying infant responsiveness during interactions (DiStefano et al., 2019), as we have done. Our results suggest the importance in future studies of only considering measurements with consistently calm behavioral states prior to onset, as well as repeating measurements at several time points. Measurement in a home rather than a laboratory environment may be more conducive to assessing naturalistic interactions accurately.

Our findings add to a small, but growing, body of literature showing differences in behavioral and neural measures of responsiveness between irritable and non‐irritable infants/children. Previous work has shown that more irritable infants demonstrate less optimal infant behavior in face‐to‐face interactions with mothers (Murray et al., 1996). Another study found lower EEG alpha power in irritable as compared to non‐irritable children with autism‐spectrum disorder (DiStefano et al., 2019). Underlying physiologic differences between irritable and non‐irritable infants, such as heart rate recovery after stress, may explain the differences in infant responsiveness between these groups (Gunning et al., 2013). Whether physiologic differences explain infant irritability/responsiveness or infant irritability/responsiveness explains physiological differences is beyond the scope of this work.

In addition, future use of our paradigm would benefit from repeated measures of child temperament to inform whether irritability is a state or a trait because irritability as a trait in young children is associated with disruptive behavioral disorders (Leibenluft & Stoddard, 2013). Although less is known about irritability in infancy and subsequent psychopathology, studies have found increased hours of infant fussing correlated with less efficient sensory processing, as well as behavior and attention difficulties at 3–8 years (DeSantis et al., 2004). Other studies have associated anger at 4 months with decreased inhibitory control at 4 years (He et al., 2010), and anger/frustration in infancy with aggression at 6–7 years (Rothbart et al., 1994). Finally, Dunn's model of sensory modulation in infants postulates that infants adapt their responses to an innate sensory neurological threshold, closer to a trait than a state (Dunn, 2007). It is unclear at this time whether this threshold may have played a role in infant neural and behavioral responses. Future studies could evaluate the infant's sensory profile in the home to help characterize the potential influence of an atypically low or high threshold on dyadic interactions.

### 4.2 Infant frontal asymmetry in non‐irritable infants may indicate more approach behaviors in the context of maternal multisensory scaffolding

In non‐irritable infants, the association between WECS and FAS measures of infant responsiveness distinctly increased for action 3 (mother smile + affectionate touch) and action 4 (mother smile + affectionate touch + emotional speech). With multisensory input, FAS shifted from negative, indicating infant withdrawal response (right asymmetry), to positive, indicating an approach response (left asymmetry).

We expected to see more positive FAS in infants across the interaction as more sensory stimuli were added to the interaction. In previous studies, infants as young as 3 months showed increased fixation times and orienting responses to visual plus touch gestures as opposed to visual gestures alone (Addabbo et al., 2015). Thus, even young infants demonstrated a more positive reaction to multisensory, as opposed to unisensory, input. Infants in the first months of life rely on multisensory redundancy for attention, learning, and discrimination (Bahrick & Lickliter, 2000; Lewkowicz, 2008). Presentation of amodal (not specific to only one sense) redundant stimuli in a multimodal context supports selective attention and eventually perceptual narrowing to relevant events in infants (Bahrick et al., 2004; Bahrick & Lickliter, 2000; Lewkowicz & Ghazanfar, 2009; Murray et al., 2016). Taken together, these findings suggest that multisensory redundancy is critical in early social and perceptual development (Purpura et al., 2017).

To our knowledge, this is the first study to specifically examine neural measures of approach in infants with maternal layered multisensory input. Our finding of a transition from negative to positive FAS in non‐irritable infants with multisensory input from the mother (actions 3 and 4) may indicate a discrepancy in behavioral expectations (incongruence) when a multisensory interaction is expected due to past experiences, and only a unisensory interaction is provided (hence negative FAS in actions 1 and 2). Multisensory development is a progressive process that is experience‐driven (Lewkowicz & Ghazanfar, 2009; Murray et al., 2016), and social interactions, particularly with mothers, are formative experiences for infants (Okabe et al., 2012; Stack & Arnold, 1998). Thus, the mother as an agent of multisensory scaffolding appears critical in the infant's development of perception, learning, and behavior (Goldstein et al., 2003; Murray, De Pascalis et al., 2016; Murray et al., 2016; Matatyaho & Gogate, 2008). By 3–4 months of age, maternal multisensory input may lead to social contingency and engagement with salient stimuli (Lewkowicz & Ghazanfar, 2009), with potentially greater approach neural patterns as observed in the current study.

### 4.3 Associations between frontal asymmetry and emotional reactivity, sensory processing, and attention

We chose the FAS measure and anticipated more positive infant FAS with additional maternally provided infant‐directed sensory stimuli because of previous neuroscientific work connecting frontal asymmetry with emotional reactivity, sensory processing, and attention. Previous work has demonstrated associations between frontal asymmetry and emotional reactivity (Coan & Allen, 2004) with more left shift (more positive FAS) indicating more approach behaviors (Anaya et al., 2021; Davidson, 1994; Hane et al., 2008). Infant EEG asymmetry seems to reflect emotional reactivity as both a state‐ and trait‐like characteristic. Frontal asymmetry reflects at least state‐like emotion. However, studies that show a longitudinal association of frontal asymmetry and later behavioral characteristics suggest that frontal asymmetry may, at least in some cases, also reflect trait‐like emotion (Coan & Allen, 2004; Fox et al., 2001; McManis et al., 2002).

In addition, ERP amplitude in frontal regions (albeit not alpha band) is a biomarker of sensory (auditory, tactile) response processing in infants (Chorna et al., 2018; Maitre et al., 2017; Therien et al., 2004). Another study found differences in FAS in children who show atypical sensory‐seeking behaviors (Damiano‐Goodwin et al., 2018), providing further evidence of the association between frontal asymmetry and sensory processing in children.

Furthermore, we based our hypothesis on the relationships between FAS and attention. Emerging executive attention networks include the prefrontal cortex in infants (Ellis et al., 2020; Posner & Rothbart, 2007). Alpha oscillations gate cortical processes by transient inhibition of cortical and subcortical signals (Jensen & Mazaheri, 2010). Differences in hemispheric inhibitory oscillations influence brain‐to‐body processing of signals and body‐to‐brain attention (Mazaheri et al., 2009). Additionally, studies support associations between FAS and attentional problems, with individuals with ADHD showing more left hemispheric cortical activity in the alpha band than controls, possibly indicating excessive approach tendency (Keune et al., 2011). Our finding that FAS transitioned from negative to positive in non‐irritable infants with increased sensory stimulation in the context of a mother–child interaction is consistent with previous work on emotional reactivity, sensory stimulus processing, and attention.

### 4.4 Maternal perception of bondedness and maternal and infant behavioral responsiveness

In our study, maternal perception of bondedness was not clearly associated with behavioral or neural patterns of infant responsiveness. Overall maternal perception of bondedness has been linked to physiological markers, including greater fMRI activation patterns in the middle frontal gyrus when a mother views her own versus an unknown infant (Wan et al., 2014). Higher maternal oxytocin levels, another physiologic marker of bondedness, are associated with maternal behaviors towards infants including positive affect and affectionate touch, among others (Bick et al., 2013; Feldman et al., 2007). In addition, mother and infant behavioral responsiveness are positively associated (Gunning et al., 2013; Kuchirko et al., 2018). Whether maternal *perception* of bondedness and maternal behaviors are equivalent is less certain. One study found a self‐reported bondedness marker was not associated with mother interactive behavior (Wan et al., 2014), while another study found that in depressed mothers, maternal perceptions of bondedness were associated with maternal attunement and attitude towards the infant during a play session (Hornstein et al., 2006). The variation among tools measuring maternal perception of bondedness and populations studied makes it challenging to conclude about associations between these perceptions and maternal behaviors and interactions. Further research is needed to elucidate the complex and nuanced relationships between maternal perception of bondedness, physiologic markers of bondedness, and both maternal and infant behavioral responsiveness.

## 5 LIMITATIONS

Our study has several limitations. First, the WECS was designed to assess both mother and infant during a short free‐play interaction. Given that the structured and mother‐led nature of our interaction could alter the mother's behaviorally coded interaction scores, we addressed this issue by limiting our coding to the infant components. Second, unmeasured differences may exist between infants who are only irritable in the moment (state) versus those who are generally irritable at baseline (trait). We have been clear throughout this paper that we can only comment on the infant state of irritable versus not irritable and not infant trait. As our study took place in a clinical laboratory setting rather than in a naturalistic home environment, we considered the potential impact of this unfamiliar environment on the state of our participants and attempted to mitigate the effect of state by averaging four trials. Our lab team is experienced with EEG acquisition in young children and has low rates of EEG data attrition for this and other studies (Chorna et al., 2019). While the continuous EEG recording and averaging of four trials in this paradigm help provide enough usable EEG data, other groups may have more EEG data attrition in young children (Kaduk et al., 2016; Michel et al., 2017). However, for this study, we had similar quality EEG data on the continuous recording for both irritable and non‐irritable infants. We know that EEG data during drowsiness may not reflect EEG data during alert periods (Noreika et al., 2020). Fortunately, only seven participants had any drowsy trials, and of these seven, 80% of their actions still had ≥2 trials to the average for that action. Thus, even for this relatively infrequent situation, our averaging of four trials per action helps account for variability in the infant state. For this study, the average number of trials per action was 3.6 without significant differences in the number of included trials between actions or groups, indicating that we were able to use most of our data (Table S3). Regarding FAS calculations, it can be difficult to compare FAS results across literature due to the different equations used. To account for this, we used the most frequently used equation to calculate FAS (FASratio), which is highly correlated with the other most frequently used equation (FASln equation) (Vincent et al., 2021).

Due to the pilot nature of this study, we were not powered to counterbalance the order of modality presentation. Thus, it is possible that increased infant responsiveness was due to the specific modalities presented in the later actions rather than the increased number of modalities. We know that the mother's face is particularly salient to infants, even shortly after birth (Field et al., 1984). Thus, we expected that this unimodal stimulation could be enough to elicit a reaction from the infant. This work, and others, suggests that infants are more responsive to multisensory than unisensory input (Addabbo et al., 2015; Vaish & Striano, 2004). This is likely because even young infants have temporal windows that allow them to integrate multiple stimuli into a single perceptual event (Bahrick, 1983; Hillock et al., 2011). Over time, these congruent events are more salient to infants, leading to perceptual narrowing for relevant events in infants(Bahrick et al., 2004; Purpura et al., 2017; Lewkowicz & Ghazanfar, 2009; Bahrick & Lickliter, 2000; Murray et al., 2016). While we suggest that these temporal windows and perceptual narrowing explain our results rather than the order of presentation of stimuli, this work could be expanded to include randomization of stimulus order presentation. Furthermore, future work with larger sample sizes could include other parental or infant variables that could impact dyadic interaction in addition to bondedness, depression, and irritability.

The binary categorization of maternal bondedness may have limited our power to detect a difference. We chose to dichotomize maternal bondedness to mirror our irritable versus non‐irritable analysis, and we used a bondedness threshold consistent with current literature (Bienfait et al., 2011; Taylor et al., 2005). Additionally, this study included only mothers and infants. Generalizability would be increased by including fathers and/or other caregivers in future iterations. Finally, the grand average correlations presented in Figures 7 and 8 only include four data points. We performed this correlation to allow us to visualize the slope of the line across the interaction. Overall, this pilot study is experimental, hypothesis‐generating, and suggests future areas of inquiry.

## 6 CONCLUSIONS AND FUTURE DIRECTIONS

We report for the first time the use of both behavioral and brain‐based measures to quantify infant responsiveness during a mother‐initiated sensory layering interaction procedure. Infant behaviorally coded positive responsiveness increased as the mother added additional sensory modalities to the interaction. Furthermore, in non‐irritable infants, neural measures of responsiveness (FAS) indicated a shift from withdrawal to approach behaviors with increased multisensory input from the mother. These findings support the validity of a neural marker of infant responsiveness that could be particularly salient in pre‐verbal infants with immature behavioral responsiveness. Given the decreased responsiveness of irritable compared to non‐irritable infants, future studies may facilitate a calm infant state, perhaps aided by a natural home environment with the use of portable EEG systems. Future work is needed to examine how extensively mother and infant neural responsiveness is connected during interactions and to use neural measures in mothers to examine maternal bondedness during dyadic interaction.

## AUTHOR CONTRIBUTIONS

MLN was responsible for study design, data collection, data analysis, and writing the initial and final drafts. AJ was responsible for study design, data collection and analysis, creating figures, and revising the manuscript. AK was responsible for study design, data analysis, and revising the manuscript. ARS was responsible for data analysis and revising the manuscript. ESN was responsible for data analysis and revising the manuscript. LR was responsible for data analysis, creating tables, and revising the manuscript. KH was responsible for data collection and revising the manuscript. NLM was responsible for study design, data collection and analysis, and drafting and revising the manuscript.

## CONFLICTS OF INTEREST STATEMENT

The authors declare no conflicts of interest.

### PEER REVIEW

The peer review history for this article is available at https://publons.com/publon/10.1002/brb3.3253

## Supporting information

Supplemental table 1 Entire interaction procedure and nomenclature
Supplemental table 2 WECS coding details
Supplemental table 3 Average numbers of trial by action and group

## Data Availability

The data that support the findings of this study are available from the corresponding author upon reasonable request.
