# Supplementary material for: Behavioral and neural measures of infant responsivity increase with maternal multisensory input in non‐irritable infants
Source: Brain Behav. 2023 Oct 2;13(11):e3253. doi: 10.1002/brb3.3253 (PMC10636412; doi:10.1002/brb3.3253)
Supplement: Supplementary file 1 — Supplemental table 1 Entire interaction procedure and nomenclature Supplemental table 2 WECS coding details Supplemental table 3 Average numbers of trial by action and group [file BRB3-13-e3253-s001.docx]

**Supplemental materials**

***Supplemental table 1***

***Entire interaction procedure and nomenclature***

| Action | 1 | B | 1 | B | 2 | B | 2 | B | 3 | B | 3 | B | 4 | B | 4 | B |
| --- | --- | --- | --- | --- | --- | --- | --- | --- | --- | --- | --- | --- | --- | --- | --- | --- |
| Length of time (seconds) | 10 | 15 | 10 | 15 | 10 | 15 | 10 | 15 | 10 | 15 | 10 | 15 | 10 | 15 | 10 | **120** |
| Run | 1 | 1 | 1 | 1 | 1 | 1 | 1 | 1 | 1 | 1 | 1 | 1 | 1 | 1 | 1 | 1 |

| Action | 1 | B | 1 | B | 2 | B | 2 | B | 3 | B | 3 | B | 4 | B | 4 | B |
| --- | --- | --- | --- | --- | --- | --- | --- | --- | --- | --- | --- | --- | --- | --- | --- | --- |
| Length of time (seconds) | 10 | 15 | 10 | 15 | 10 | 15 | 10 | 15 | 10 | 15 | 10 | 15 | 10 | 15 | 10 | 15 |
| Run | 2 | 2 | 2 | 2 | 2 | 2 | 2 | 2 | 2 | 2 | 2 | 2 | 2 | 2 | 2 | 2 |

B=break

Run = 8 actions

Segment = EEG data acquired from trial

***Supplemental table 2***

***WECS coding details***

| **WECS category** | **Score = 0** | **Score = 1** | **Score = 2** | **Score = 3** |
| --- | --- | --- | --- | --- |
| **Facial expressiveness** | **N/A** | **Negative**  No response or continual cry | **Variable/mixed**  Intermittent fuss | **Positive**  Smile, wide mouth, vowel sound, or laugh |
| **Sensitivity** | **Asleep**  Sleeping | **Rarely attentive**  Awake but no interest | **Variable**  Loses interest | **Follows mother/positive response**  Maintains interest |
| **Vocal communication**  (Also includes behavioral responsiveness in preverbal infants (Hane et al., 2018)) | **N/A** | **Negative**  Not consolable or irritable but can console | **Variable**  Irritable when approached or Neither happy nor unhappy | **Positive**  Happy and smiling |

***Supplemental table 3***

***Average numbers of trial by action and group***

|  | mean | std |  | mean | std |  | mean | std |  |  |
| --- | --- | --- | --- | --- | --- | --- | --- | --- | --- | --- |
| General | 3.6 | 0.42 | action1 | 3.2 | 0.58 | irr | 3.1 | 0.47 | t=0.63 | p=0.545 |
|  |  |  |  |  |  | non irr | 3.3 | 0.69 |  |  |
|  |  |  | action2 | 3.8 | 0.38 | irr | 3.8 | 0.38 | t=0.15 | p=0.882 |
|  |  |  |  |  |  | non irr | 3.8 | 0.38 |  |  |
|  |  |  | action3 | 3.7 | 0.35 | irr | 3.72 | 0.33 | t=0.53 | p=0.608 |
|  |  |  |  |  |  | non irr | 3.68 | 0.37 |  |  |
|  |  |  | action4 | 3.7 | 0.35 | irr | 3.7 | 0.35 | t=-0.3 | p=0.763 |
|  |  |  |  |  |  | non irr | 3.7 | 0.35 |  |  |
|  |  |  |  |  |  |  |  |  |  |  |
|  |  |  | action | F=1.98 | p=0.157 |  |  |  |  |  |
